# Development and Testing of an Exergaming and Artmaking Program to Enhance Cognitive and Physical Function in Older Adults: Results of a Community-Based Pilot Study

**DOI:** 10.2196/73555

**Published:** 2025-08-29

**Authors:** Nikhil Satchidanand, Mia Lawrence, Sameer Jhaveri, Sara Opalka-Satchidanand

**Affiliations:** 1 Department of Internal Medicine, University at Buffalo, State University of New York, DK Miller Building Room C209, 462 Grider Street, Buffalo, NY, 14215, United States, 1 7168162884; 2 Department of Internal Medicine, University of Pittsburgh, Pittsburgh, PA, United States; 3 Fine Art Miracles, Pittsburgh, PA, United States

**Keywords:** exercise, aging, art, community, exergaming, older adults, social support

## Abstract

**Background:**

Group-based recreational activities, including physical activity and art-making, can help older adults preserve or even improve key functional outcomes essential to successful aging, while also fostering social support. Community-university partnerships can promote successful aging by facilitating the design and implementation of evidence-based, relevant, and impactful programs that better meet the needs of older adults in our community.

**Objective:**

This study aimed to leverage a community-integrated approach to develop and test a recreation-based exergaming and art-making program on improving cognitive and physical functional outcomes in older adults.

**Methods:**

Working with the Buffalo-Niagara YMCA, we developed the “Movers and Makers Club,” a group-based recreation program to improve cognitive and physical function in aging. We recruited adults, ≥65 years, to participate in weekly group-based SMARTfit exergaming and artmaking during the 12-week program. The program was divided into 2 phases. During the design phase, we sought direct feedback from older adults in the community regarding the appeal and usability of proposed exergaming activities and art projects. In the impact phase, we administered a 1-group, 12-week pilot study to test the effects of group-based SMARTfit exergaming and visual artmaking on performance on the Trail-Making Test and Stroop Color and Word Test. In addition, other functional assessments included a 4-Stage Balance Test, 30-second Sit-to-Stand test, and a 6-Minute Walk Test. Key participant-reported outcomes were also collected on the appeal, enjoyability, and usability of program activities.

**Results:**

In all, 17 older adults (mean age 73.80, SD 4.10 y) enrolled in the program between 2 YMCA branches: one representing the City of Buffalo and one, a local suburb. At the end point, all 17 participants completed at least 9 out of 12 sessions. Participants reported high ratings of enjoyment and satisfaction with their experience. They also reported that their comfort level while exercising, maintaining their balance, and walking had improved. Participants also found their comfort level with doing art, and their interest in trying new artistic pursuits increased. In addition, performance on the Trail-Making Test and Stroop Color and Word Test improved from baseline to end point (*P*<.001). We also observed improvements in 1-leg balance, 30-second Sit-to-Stand test performance, and 6-Minute Walk Test distance (*P*<.001).

**Conclusions:**

Our group-based recreation program, delivering SMARTfit exergaming and guided art-making to older adults, improved cognitive and physical functions important to successful aging. In addition, our approach of seeking direct feedback from older adults, in developing the program, produced a highly enjoyable and appealing experience that was age- and ability-appropriate. Our future programs will integrate formal assessments of key psychosocial factors that may influence both the implementation and functional impacts of the “Movers and Makers” curriculum.

## Introduction

Preserving cognitive and physical function is critical to successful aging [1-3]. Recreational activities, such as group exercise and visual artmaking, offer older adults an enjoyable experience while simultaneously helping them maintain function, independence, and quality of life [4-14]. These activities also often provide a social experience and have the potential to enhance functional capacity while promoting social support [8,10,14,15]. In a review by Ali et al [6] that examined the effects of cognitive-motor exercise on cognition and physical function, they found small to medium effect sizes ranging from 0.19 to 0.56 for balance, dual-task cost, gait speed, attention, memory, executive function, and global cognition. Similarly, in our previous YMCA-based program, we found that 12 weeks of group-based cognitive-motor exercise, using the SMARTfit Cognitive-Motor Exercise System (Ventura, CA), was associated with significant improvements in attention, task-switching, and interference inhibition as assessed by the Trail Making Test (TMT) and Stroop Color and Word Test (SCWT) [12]. Overall, the evidence is encouraging that dual-task exercise can help older adults preserve key physical and cognitive functions critical to maintaining independence and quality of life, in a setting that fosters social support [6,16,17].

Recent studies have sought to characterize the potential of visual artmaking as a therapy to enhance functional capacity in aging. In a review by Masika et al [4], visual artmaking, including painting, drawing, and craft-based activities, was shown to improve cognitive function among older adults with mild cognitive impairment (MCI) [4]. In this review, visual artmaking was also associated with lower depression and anxiety scores among cognitively impaired and cognitively intact older adults. Programs that include socialization, art education, reminiscence, art processing and analysis, or art sharing were more likely to enhance cognitive function [4]. For instance, older adults in a nursing home setting who participated in a group-based ceramic painting program, which encouraged socialization, art sharing, and social support, demonstrated improved scores on the Mini-Mental State Examination from before to after the program [9].

Current evidence shows that separate, group-based, dual-task exercise and art-making can help maintain or even improve cognitive and physical function in aging [4,11-13,15,18-20]. A novel community-based recreation program that brings these activities together while encouraging socialization will promote participation and foster social support, while improving functions essential to successful aging. We established a collaboration with the Buffalo-Niagara YMCA to leverage the expertise and commitment of this community organization with demonstrated successes in serving the needs of older adults. This, in combination with our dedication to exploring evidence-based, relevant, and functional outcomes, allowed us to design and implement a series of novel, group-based programs to support the health and function of older adults in our community.

The Movers and Makers Club was developed from this community-university partnership. Our overarching aim was to identify ways in which our team can effectively integrate combined group exergaming and artmaking into diverse settings, which will improve outcomes in older adults. Our study was also designed to explore the enjoyability, appeal, and usability of program activities. The insights from this pilot have helped optimize program elements to create an enjoyable and impactful community-based program. To our knowledge, this is one of the first recreation-based programs that combines group dual-task exergaming with visual artmaking to explore their effects on cognition and physical function in older adults in a community setting.

## Methods

### Participants and Recruitment

Our approach to recruitment and participant inclusion was derived from our previous work [12]. Adults, 65 years or older, were recruited from nearby senior centers, libraries, and other community organizations using recruitment flyers. Older adults interested in participating contacted the YMCA by telephone to schedule a consent and screening session. Screening for eligibility included (1) cognitive assessment (Montreal Cognitive Assessment [MoCA]), (2) readiness (Physical Activity Readiness Questionnaire for Older Adults), and (3) capacity to provide consent (University of California Brief Assessment of Capacity to Consent). Participants were provided details about the study before enrollment, and informed consent was obtained.

Participants were included if they (1) were >65 years of age, (2) had no more than MCI (MoCA score ≥24 points), (3) had no contraindications to exercise guided by the American College of Sports Medicine, and (4) were fluent in English. Potential participants were not included if (1) they had a condition that would prevent safe participation in the exercise, as determined by the Physical Activity Readiness Questionnaire for Older Adults, (2) severe neurological disease, (3) severe psychiatric illness, (4) likelihood of withdrawing from the study due to severe illness or a life expectancy of <6 months, (5) they had a lower or upper limb amputation, and (6) had greater than MCI (MoCA score <24 points).

### Study Phases

#### Design Phase

The design phase introduced community members to the SMARTfit training and art-making projects that were then integrated into the impact phase. The purpose was to garner direct feedback from older adults before testing the activities. This was to ensure an enjoyable, age- and ability-appropriate recreational experience.

The dual-task training program was delivered using the SMARTfit Cognitive-Motor Exercise System (SMARTfit). SMARTfit is an engaging exergaming platform used to facilitate dual-task training. This system has a series of touch-sensitive LED arrays attached to wall-mounted, high-strength composite panels. The LED arrays are programmed to deliver several cognitive games, including memory, alphabet, counting, and pattern recognition. Adding a simultaneous physical task, such as stepping in place, creates a cognitive-motor challenge, allowing hundreds of combined tasks to be executed. Participants can play various games by striking the individual targets with their hands or by hitting them with other objects such as “pool noodles,” drumsticks, or tennis balls. The SMARTfit system facilitates dual-task training while delivering an experience that is fun and engaging [12].

We conducted one 45-minute small group session at each of the 2 YMCA branches. In all, 12 participants completed several cognitive tasks, including a memory game, chase the target, addition, and subtraction. These tasks were paired with stepping in place, walking to and from the SMARTfit board, striking the targets with “pool noodles,” or lightly punching the targets (with boxing gloves on). Different game durations were tested, ranging from 30 seconds to 2 minutes.

Proposed art-making projects were also presented to older community members to gather their feedback and preferences regarding media type, project complexity, and the overall appeal of each project. A total of 11 older adults completed this phase at the Ken-Ton Family YMCA (KTY) branch. One 40-minute session was administered to introduce the art-making portion of the program. As shown in Table 1, example projects involving several media were presented along with a brief description of the project. This was meant to enhance participant understanding of the art projects and ensure a wide variety of projects were included, thus giving participants many opportunities to find enjoyable, rewarding projects at which they could succeed.

**Table 1.** Example art projects with corresponding inspiring artists used to deliver the art-making component of the Movers and Makers program.

Artist and inspiration	Project
David Hockney	Landscapes: oil pastel
Lascaux, France	Cave paintings: soft pastel
Terrance Osborne	New Orleans stylized painting: acrylic
Henri Matisse	Cut-paper collage
Yayoi Kusama	Mushroom art: acrylic
Georgia O’Keeffe	Landscape: watercolor

#### Impact Phase

The impact phase consisted of a 1-group intervention administered at 2 branches within the Buffalo-Niagara YMCA system. We assessed changes in cognition and physical function associated with 12 weeks of group-based SMARTfit training and guided artmaking. In all, 17 participants completed the program between the 2 YMCA branches. A total of 8 participants were enrolled at the William-Emslie Family YMCA (WEY), a city-based branch with a membership that is predominantly Black. In all, 9 participants were enrolled at the KTY, a branch that has a membership that is predominantly White. For both YMCA branches, the principal investigator (NS) monitored the trial conduct monthly and determined criteria for stopping the program early for any participant, which included participant request or self-disclosure of a new diagnosis that would meet exclusion criteria.

Program participants completed one 60-minute, group-based, exergaming session and one 60-minute guided group art-making session per week for 12 weeks. SMARTfit and art sessions were facilitated by trained and experienced instructors. The SMARTfit program was created by the principal investigator (NS), while the group art-making program was developed by the cocreator and art instructor (SO-S) for the “Movers and Makers Club.”

### Description of the SMARTfit Program

At each YMCA branch, participants completed the exergaming training in groups of 6-8 individuals. Sessions began with a 3-minute warm-up including standing stretching, walking in place, and light standing calisthenics. The SMARTfit instructor then introduced the dual-task training games for that session, guiding the group through each game. Participants completed the same training games of the same duration and order. As illustrated in Table 2, several individual and team (involving 2 participants working together) games were played.

**Table 2.** Example SMARTfit dual-task training games with focus areas, used to deliver the 12-week, 1-group, dual-task exercise program.

Name of game	Focus areas	Individual	Team
Subtraction with drumstick tap	Processing speed, eye-hand coordination	✓	—^a^
Pattern replication with medicine ball tap	Working memory, attention, strength	✓	—
Knock-out for speed with punching	Eye-hand coordination, processing speed	✓	—
Kicking with one-leg stand (assisted or unassisted)	Strength, attention	✓	—
Left, right, or both hands	Attention, processing speed, eye-hand coordination	—	✓
Addition with side-step (alternating)	Balance, processing speed	—	✓
Chase the color with ball toss and catch (alternating)	Eye-hand coordination, attention	—	✓
Counting with medicine ball carry (alternating)	Attention, strength, processing speed	—	✓
Trail-making with ball bounce pass (alternating)	Eye-hand coordination, attention, processing speed	—	✓

a Not applicable.

Games coincided with specific functional domains, including balance, muscle strength, hand-eye coordination, attention, and memory. These domains were identified and tested in the development of our previous trial [12]. A variety of physical movements added a dual-task dimension to the cognitive training (eg, stance changes, sit-to-stand, or patterned stepping). The games were 30 seconds to 2 minutes, and each training session allowed 4-7 different games to be completed. From our previous study, we estimate that each participant accumulated 8-10 minutes of sedentary time while transitioning from one game to the next. Each session concluded with 3 minutes of cool-down. During training, speed and accuracy of the response to each challenge were emphasized. In addition, participants were instructed not to help each other while performing the cognitive challenges. Instructors monitored participant safety throughout each training session.

### Description of the Artmaking Program

Participants completed 1 hour of guided artmaking for 12 weeks, in small groups of up to 12 individuals, allowing for sufficient one-on-one instruction time. Art sessions were completed before or after the SMARTfit training, based on each participant’s schedule.

Art sessions began with an introduction to the project, a brief description of the inspiring artist or art movement and history, and an explanation of the technique and media being used. Participants were provided with all the materials required, instructions for completing the project, and several complete examples of each project for reference and inspiration. In addition, templates, pre-made project elements (eg, pre-cut paper for collages), and other supportive materials were given to participants to promote success and ensure completion of each project in a timely manner.

A wide array of projects was selected to expose participants to many different media and techniques, while also ensuring participants completed several projects that were enjoyable and rewarding. Individual expression within each project was encouraged, as were artwork-sharing and discussion. The art instructor (SO-S) remained focused on supporting, guiding, and inspiring participants as they worked on their projects.

### Outcomes Assessment

#### Participant-Reported Outcomes

A brief survey instrument was created for this project to explore participants’ experiences in the program. This instrument was assessed for acceptability and ease of understanding in 10 older community members before using it for this program. Themes explored included participant interest, appreciation, comfort level, and confidence with each activity after completion of the Movers and Makers program. In addition, we asked participants to evaluate the quality of instruction, their overall enjoyment, and satisfaction with each program component. Participants were also asked to comment on any changes they thought would improve their experience. This information was used to design an expanded program involving several additional YMCAs and to recruit sufficient participants to more deeply assess changes in key functional outcomes.

#### Cognitive and Physical Function

Outcome assessment methods were adapted from our previously published trial [12]. Assessors administered the battery of instruments in an office or meeting room in private, to allow sufficient privacy to ensure participants were able to concentrate on the tasks at hand. The assessors were blinded to baseline performance at the time of completion of the end point assessment, having no access to baseline scores. The TMT and SCWT were administered at baseline and end point. The TMT assesses task-switching, visual attention, and memory. This test has 2 parts, TMT-A (rote memory) and TMT-B (executive functioning). The participant draws a line to connect 24 consecutive circles containing numbers and letters randomly arranged on a sheet of paper, without lifting the pen tip off the paper [21]. The TMT-A section has numbers, while the TMT-B has both numbers and letters, requiring the participant to alternate between a number and a letter in ascending order. The total score for each section is based on the time (in seconds) needed to complete each section. The TMT was administered on paper, once at baseline and once at end point, using standard procedures including 1 sample trial and 1 test trial [21].

The SCWT assesses interference inhibition and working memory. The participant being assessed is instructed to read aloud 3 different tables, one at a time, as quickly as possible. The first 2 tables are congruent conditions requiring the participant to read names of colors printed in black ink (word condition) and name different blocks of colors (color condition). The third condition is incongruent (color-word), containing color names printed in a different colored ink (eg, the word “red” printed in “blue” ink). The participant must name the color of the ink, not read the word. The score earned is based on the number of correct answers given in each condition within 45 seconds [22]. The SCWT was administered individually, with the participant accessing each table one at a time, at baseline and end point. A sample of each table was presented to anchor participants to the assessment.

Physical function was measured using a modified 4-Stage Balance Test (FSBT), a 6-Minute Walk Test (6MWT), and a 30-second Sit-to-Stand test (30STS). The FSBT included side-by-side, tandem, and left and right one-leg stands. These tests were administered individually in a private room by a trained assessor. The order of assessment was (1) FSBT: side-by-side, tandem, one-leg (timed for 10 seconds each); (2) 6MWT; and (3) 30STS. A higher score indicates better performance on each functional measure.

### Data Management

Study data, both outcomes and safety, were collected by the principal investigator (NS) and project coordinator using collection forms and guided by the standard procedures for each assessment (eg, TMT and SCWT) that were described in the Methods section. Quantitative data were entered into a Microsoft Excel database weekly by a trained research assistant. Biweekly data quality checks were performed by the principal investigator (NS) by reviewing the paper data collection form and entered data. The password-protected Excel file was stored on a password-protected computer in the principal investigator’s (NS) office. Safety data were reviewed by the principal investigator (NS) and project coordinator weekly, after completion of each training session.

### Statistical Methods

First, measures of central tendency (mean, median, and mode) were calculated to assess continuous variables, while categorical and questionnaire data were examined using frequencies. To assess participants’ experiences in the Movers and Makers program, responses to the survey items were examined as proportions of the highest score on each response. In addition, free-text responses were examined for commonly occurring themes. For both the quantitative and qualitative outcomes, we found no missing data.

A preliminary sample size calculation revealed that to detect a large effect size (0.50) in change in scores on the TMT, with a statistical power of 0.80, a total of 26 participants needed to be enrolled. Due to the low sample size and nonnormal distribution, median and IQR were calculated to explore pre- and postprogram performance on the TMT, SCWT, FSBT, 30STS, and 6MWT. Wilcoxon Signed Rank Tests were conducted to examine changes in performance on each test. In addition, the predicted color-word score (Pcw) was calculated based on scores on the 2 congruent conditions, using the following calculation:

Pcw=45/{((45×W)+(45×C))/(W×C)}

The interference score (IS) was generated by subtracting the Pcw from the number of items correctly named in the Color-Word condition. As guided by Ivnik et al [23], a lower IS represents more difficulty inhibiting cognitive interference. Pre- to postprogram change in the IS was also assessed using a Wilcoxon Signed Rank test.

### Ethical Considerations

The participants’ protections in this study were reviewed and approved by the University at Buffalo institutional review board (STUDY00003525). All participants provided informed consent. Participants reviewed the consent form independently in a private office, allowing them sufficient time to examine all elements of the study thoroughly. Time was then provided for participants to ask questions or voice concerns to the researcher obtaining consent. Participants were compensated with a US $20 prepaid gift card to complete the baseline and end point assessments. In addition, the University of California Brief Assessment of Capacity to Consent was used to confirm that participants fully understood the consent form and the study procedures. Study data are being stored deidentified, with each participant assigned a unique identification number. The key that links participants to their data is being stored as a separate password-protected file on a password-protected computer in the principal investigator’s (NS) office.

## Results

### Sample Characteristics

In all, 12 older adults completed the SMARTfit user groups in the design phase, consisting of 11 females and 1 male with a mean age of 71.28 (SD 2.54) years. A total of 11 older adults completed the introductory art session, including 10 females and 1 male. The mean age in this group equaled 74.43 (SD 2.67) years.

**Figure 1.**
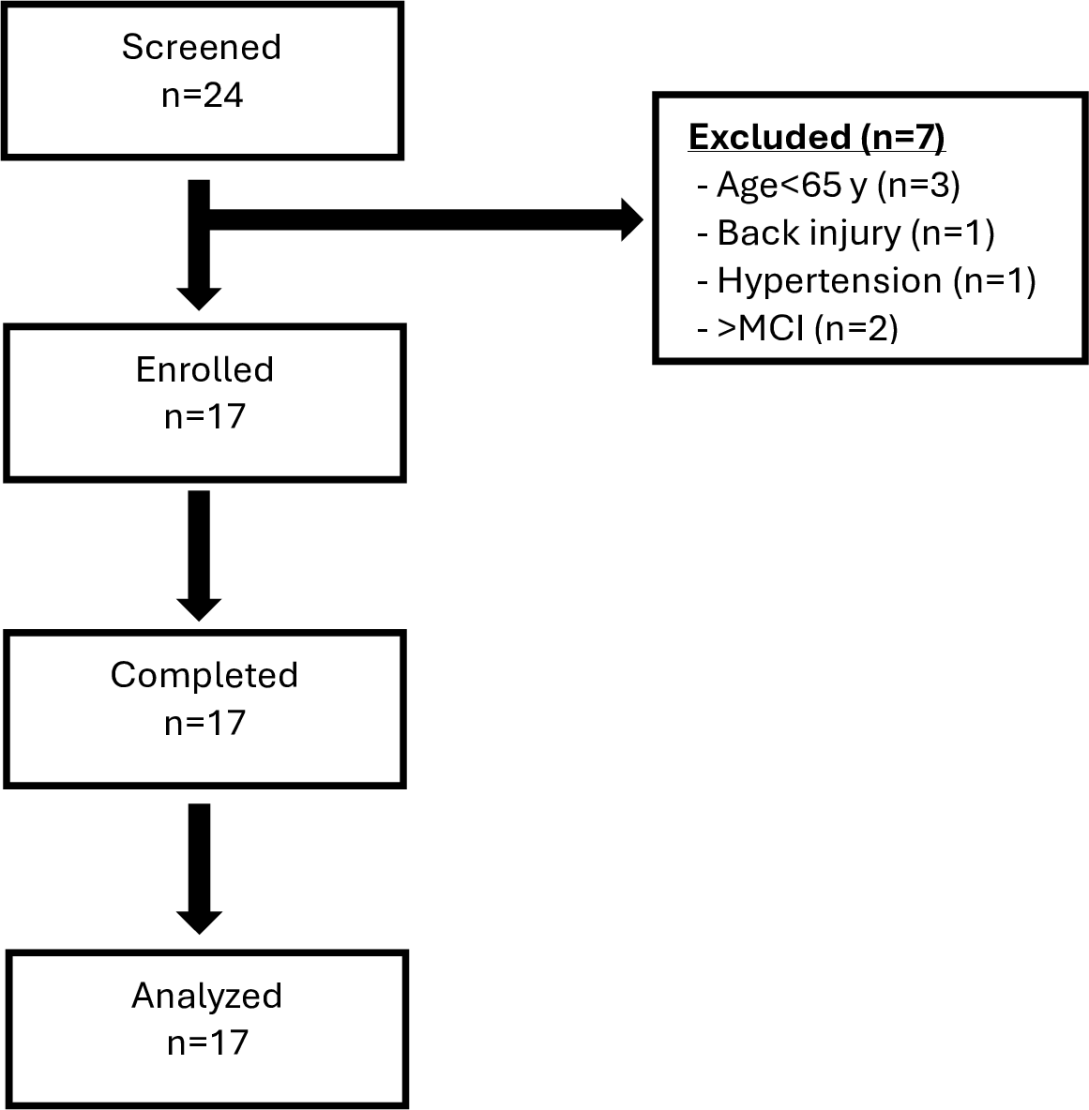
Participant flow diagram

A total of 24 older adults volunteered to participate in the impact phase and were screened for eligibility, resulting in 17 being enrolled. Please refer to Figure 1 for participant flow through the research process. Exclusion was based on not meeting minimum age (n=3), a previous unresolved back injury (n=1), uncontrolled hypertension (n=1), and cognitive impairment beyond mild (n=2).

In all, 9 participants were enrolled at the KTY and 8 participants were enrolled at the WEY. At the end point, all 17 enrolled participants were “completers,” defined as participating in at least 9 of the 12 sessions, and 5 participants completed all 12 sessions. Among the participants at the KTY, the mean age equaled 72.30 (SD 3.60) years and the mean age at the WEY equaled 74.13 (SD 4.28) years; these means were not found to be statistically significantly different (*P*=.34). In addition, all participants self-reported as female and 94% (16/17) reported being retired, while 6% (1/17) reported working part time. There were no differences between the 2 YMCA branches by employment status (*P*=.92).

### Participant Experience

Overall, participants favorably rated their experiences in the Movers and Makers program. In all, 100% (N=17) of participants responded with “Very Enjoyable” when asked to rate their enjoyment on a scale of 1‐5. In addition, 100% (N=17) reported they were “Highly Satisfied” with their overall experience. When they reflected on the social aspect of the program, 100% (N=17) of participants reported that it enhanced the program and their experience. One participant at the KTY stated that being in Movers and Makers helped her try new activities and meet new people. She said,

*I thought the exercise and art would be fun, which is why I joined the program. The SMARTfit exercises were unique, and I enjoyed the games more than regular group exercises I tried before. I didn’t realize how much fun we would have as a group. Playing SMARTfit together and sharing our art really helped me make some new friends that I don’t think I would have met in another class.*

When asked about the perceived benefits of the SMARTfit training, 100% (N=17) of participants reported that their experience improved their comfort level with physical activity and made them feel more capable while walking, maintaining their balance, and avoiding falls. In addition, 100% (N=17) of participants reported their interest in trying new physical activities increased after the program. One participant at the WEY reported that being part of the Movers and Makers “Club” helped her realize her capabilities. She stated that,

*Being on the M&M team made me see I am capable of much more than I realized. I used to be afraid of walking in large crowds, like at the mall. I thought I was walking so (slow) and would get walked right over. But now I see I can do it, even with a bad hip. I am definitely more comfortable and confident in public.*

Similarly, when asked about their experiences in the group artmaking, 100% (N=17) of participants had favorable responses. They reported that participation enhanced their appreciation for art, improved their comfort and confidence when doing art, and piqued their interest in trying new creative endeavors. One participant at the KTY reported that she has always found art to be intimidating. She said,

*Art always intimidated me, even as a kid. Being in the Movers and Makers was a really positive experience for me. Sara took the fear and intimidation away and made it really fun. I realize I don’t have to be perfect at it and I can improve even at my age.*

Participants were also asked to describe their experiences in 1 word, for both the SMARTfit sessions and the art classes. Several keywords were associated. The SMARTfit sessions were described as “enjoyable,” “energizing,” “fun,” “engaging,” and “beneficial.” Similarly, the artmaking was described as “interesting,” “relaxing,” “engaging,” “inspiring,” and “relatable.” Overall, all participants in the program indicated their experience was rewarding and enjoyable.

We also asked about the quality of instruction for both the SMARTfit training and art. The SMARTfit instructors were characterized as “supportive,” “encouraging,” and “well-prepared for each class.” Participants reported feeling safe while exercising in their groups and that their instructor demonstrated enthusiasm for teaching. Similarly, participants reported that the art instructor was well-prepared for each lesson, she provided clear, easy-to-follow instructions, and was “supportive” and “encouraging.” In addition, participants felt the art instructor demonstrated enthusiasm for teaching and made the lessons interesting. One participant at the WEY found that exercising with other women, with guidance from the instructor, was encouraging. She said,

*I was scared to do the exercise part of the program because I had not exercised in many years and I can’t move too (quick) now. But, on the first day I saw a few other women moving like I do. Our instructor was so encouraging and helped us stay positive.*

Participants were also asked to describe any challenges they had with the program or any changes they would recommend. Furthermore, 3 participants reported that they felt some of the physical movements during the SMARTfit sessions were too difficult for them and that they felt discouraged at first.

### Performance Outcomes

### Cognition

The MoCA was administered before beginning the program for screening purposes, to identify potential participants with cognitive impairment beyond mild. At baseline, the median MoCA score was 26.00 (IQR 25.00-27.00). For the cognitive assessments, the scores are reported as median (IQR). Median baseline performance on the TMT-A improved from 43.10 (IQR 42.14-45.02) seconds to 41.00 (IQR 39.56-41.99) seconds (W=1.00, *P*<.001, *r*=−1.00; Table 3). The score on the TMT-B also improved from baseline to end point, with a median baseline of 140.30 (IQR 137.27-145.29) seconds to an end point median of 137.80 (IQR 134.07-141.38) seconds (W=1.00, *P*<.001, *r*=−1.02; Table 3). In addition, performance on the color condition and word condition of the SCWT improved from baseline to the end point (*P*<.05; Table 3). Similarly, the incongruent (Color-Word) condition of the SCWT also improved. At baseline, the median equaled 32.50 (IQR 30.00-34.00) seconds, and at end point, the median equaled 35.50 (IQR 33.00-36.50) seconds (W=1.00, *P*<.001, *r*=0.88; Table 3). The median IS of the SCWT also improved significantly from −4.50 (IQR –5.70 to –3.53) to −1.90 (IQR –3.50 to –1.71; *P*<.001; Table 3).

Physical function also improved from baseline to end point. Median right leg balance at baseline was 7.39 (IQR 6.49-8.95) seconds and improved to 10.00 (IQR 8.98-10.00) seconds (W=0.00, *P*<.001, *r*=1.00) (Table 3). Median left leg balance improved from 7.21 (IQR 6.89-8.60) seconds to 9.25 (IQR 7.86-10.00) seconds (W=0.00, *P*<.001, *r*=1.00; Table 3). Performance on the side-by-side stand and tandem stand did not improve. Improvement was observed in the 30STS, with a baseline median score of 16.00 (IQR 15.00-17.25) completed stands increasing to an end point median of 19.00 (IQR 18.00-22.00) completed stands (W=0, *P*<.001, *r*=0.90; Table 3). Performance on the 6MWT also improved from a median of 547.50 (IQR 497.75-571.75) m to 566.70 (IQR 522.00-583.25) m (W=2.00, *P*<.001, *r*=0.92; Table 3).

**Table 3.** Quantitative results of the Wilcoxon signed-rank tests for baseline to end point comparison of median cognitive and physical functional performance, Wilcoxon W, *z* score, and effect size for the one-group dual-task exercise program.

Outcome	Median score (IQR)	96.91% CI	W	*P* value	*z* score	Effect size (r)
TMT A^a^^,^^b^			1.00	.02	–4.32	–1.00
Baseline	43.10 (42.14 to 45.02)	42.15 to 45.01				
End point	41.00 (39.56 to 41.99)	39.67 to 41.98				
TMT B^c^^,^^d^			1.00	<.001	–4.33	–1.02
Baseline	140.30 (137.22 to 145.29)	137.27 to 145.09				
End point	137.80 (133.86 to 141.51)	134.07 to 141.38				
Stroop Color^b^^,e^			0.00	.003	2.95	0.90
Baseline	73.50 (70.00 to 75.00)	71.00 to 75.00				
End point	75.00 (73.00 to 76.00)	73.00 to 76.00				
Stroop Word^b^^,^^e^			12.50	.022	2.31	0.64
Baseline	74.00 (72.00 to 76.00)	72.00 to 76.00				
End point	75.00 (73.00 to 77.00)	73.00 to 77.00				
Stroop Color – Word^e^^,^^d^			1.00	<.001	3.72	0.88
Baseline	32.50 (30.00 to 34.00)	30.00 to 34.00				
End point	35.50 (33.00 to 36.00)	33.00 to 36.50				
Stroop Interference^d^^,f^			0.00	<.001	4.48	1.05
Baseline	–4.50 (–5.70 to –3.53)	–5.75 to –3.25				
End point	–1.90 (–3.50 to –1.71)	–3.24 to –0.99				
FSBT Side-By-Side^g^^,^^j^			—	—	—	—
Baseline	10.00 (10.00 to 10.00)	10.00 to 10.00				
End point	10.00 (10.00 to 10.00)	10.00 to 10.00				
FSBT Tandem^g^^,^^j^			—	—	—	—
Baseline	10.00 (10.00 to 10.00)	10.00 to 10.00				
End point	10.00 (10.00 to 10.00)	10.00 to 10.00				
FSBT-Left^g^^,^^d^			0.00	<.001	4.17	1.00
Baseline	7.21 (6.89 to 8.60)	6.91 to 8.42				
End point	9.25 (7.86 to 10.00)	7.88 to 10.00				
FSBT-Right^g^^,^^d^			0.00	<.001	4.01	1.04
Baseline	7.39 (6.49 to 8.95)	6.59 to 8.88				
End point	10.00 (8.98 to 10.00)	9.02 to 10.00				
30STS^h^^,^^d^			0.00	<.001	3.51	0.90
Baseline	16.00 (15.00 to 17.25)	15.00 to 17.00				
End point	19.00 (18.00 to 22.00)	18.00 to 22.00				
6MWT^i^^,^^d^			2.00	<.001	3.62	0.92
Baseline	547.50 (497.75 to 571.75)	502.00 to 571.00				
End point	563.50 (522.00 to 583.25)	523.00 to 582.00				

a TMT A: Trail-Making Test A.

b Difference is significant at the *P*<.05 level.

c TMT B: Trail-Making Test B.

d Difference is significant at the *P*<.001 level.

e Stroop Color and Word Test.

f Stroop Interference score.

g FSBT: 4-Stage Balance Test.

h 30STS: 30-second Sit-to-Stand test.

i 6MWT: 6-Minute Walk Test.

j No difference: not applicable.

## Discussion

### Principal Results

Overall, our participants reported high levels of enjoyment in the SMARTfit training and group artmaking. Previous studies demonstrate that positive affective states during exercise are associated with higher levels of adherence. In an umbrella review by Collado-Mateo et al [24], enjoyment is identified as an important predictor of exercise adherence. Enjoyable recreational experiences for older adults can substantially impact their willingness to maintain participation over time [24]. Woolley and Fishbach [25] describe enjoyment as an immediate reward that can lead to better persistence, versus the delayed reward of improved health or function. Enjoyment, then, is likely an important influence on both adoption of and adherence to health-related activities, including group exercise and art making. Our program focused on gathering input directly from older adults to identify the characteristics that are optimally appealing and enjoyable. While we could not find studies that directly investigated this phenomenon in group-based visual artmaking as an interventional therapy, it stands to reason that a similar underlying mechanism is at play. Simply, people are more likely to adopt activities they find enjoyable.

Participants reported several perceived benefits of the Movers and Makers program, informing the development of future, larger-scale community-based initiatives. Perceived benefits included increased comfort with exercise and artmaking, more physical and artistic confidence, and an increased interest in trying new activities. While we did not directly measure self-efficacy and self-competence during this small pilot, our findings are encouraging that participation in a community-based recreation program can enhance older adults’ beliefs in their capacity to accomplish exercise- or art-based activities, thus encouraging future participation [26]. Our future studies will focus on formally assessing these key constructs in the context of delivering the Movers and Makers curriculum.

Insights from participants have helped us strengthen our methods as we expand our program. Our qualitative results demonstrate that specific attributes of our program may have influenced the overall positive attitudes reported by participants. In addition to creating an enjoyable experience and providing opportunities to enhance self-efficacy and self-competence, the group-based nature was positively received by all participants. This hints at the important role of social support in promoting long-term adherence to health-related activities and ultimately in improving functional capacity in aging. Exercise-based programs that promote social connectedness are more successful at maintaining participation in the longer term [27]. We observed this among participants where words of encouragement and positive peer feedback were commonplace, and 100% (N=17) of participants enrolled completed at least 9 of 12 sessions. It is reasonable to believe that this overall positive, supportive environment was associated with a high adherence rate and overall favorable attitudes about the Movers and Makers program.

Specific cognitive outcomes showed modest improvements with 12 weeks of participation. Specifically, the TMT-B and color-word conditions of the SCWT improved, with effect sizes ranging from 0.90 to 1.10. While the changes in baseline to end point medians are small, it is encouraging that specific cognitive domains, including attention, task-switching, and interference inhibition, can be enhanced in the context of a community-based group recreation program. Previous evidence shows that these specific cognitive domains often decline with advancing age [28,29]. Development and validation of practical and appealing strategies to preserve or enhance key cognitive functions is essential to maintaining independence. Community-based programming increases the reach of interventions and provides an opportunity to deliver evidence-based therapies to those who need them while promoting social support [30,31].

We found the median IS of the SCWT improved, a score that can be derived using different methods found in the literature. Among studies that have reported an IS, most have used the equation developed by Golden in 1978 [32]. One systematic review that compared studies that evaluated indirect scores found that of 27 studies, 16 used Golden’s equation [32]. In addition, a review [22] found that 17 out of 21 studies relied on a 45-second time interval, as we did in this study. In all, 16 of these studies reported results from Golden’s standardized formula. Given our previous experience using the Golden equation [12], we elected to report it as part of our findings.

Similar to our previous work [12], we found that the SCWT IS improved after 12 weeks. However, the score remained negative, showing that despite the significant improvement, there may be lingering impairments in interference inhibition. As we stated in our previous study, the persistence of a negative IS at the end point is reasonable when we consider a relatively short training duration of 12 weeks combined with a lower weekly training volume. We anticipate that a longer duration study in combination with higher training volume would result in greater improvements in interference inhibition [12]. Key physical functional outcomes improved with participation. One-leg balance improved, reaching the maximum score of 10 seconds for the right leg and 9.25 seconds for the left leg. Improvement in 1-leg balance is noteworthy given that the ability to stand on 1 leg for at least 10 seconds is independently associated with reduced all-cause mortality and adds prognostic value for mortality beyond age, sex, anthropometric, and clinical factors [33].

On the 6MWT, we observed that the end point median value of 563.50 (IQR 522.00-583.00) m is only slightly above the expected norm of similarly aged females (mean 560, SD 49 m) as revealed in a review by Bohannon [34]. These findings are promising that walking speed can be improved in older adults with just once weekly exposure to activity. However, the exact dose of this type of dual-task training that optimizes walking ability is still unclear.

The improvements we observed in balance, walking, and lower limb strength are also of note because each is an important predictor of fall risk in aging [35-37]. We have shown that a socially engaging, fun recreation program can help reduce fall risk by enhancing key physical functional outcomes. Importantly, the Movers and Makers program was not specifically designed to reduce the risk of falling in this population; however, our results are interesting. Our future efforts will focus more closely on testing the impact of the Movers and Makers curriculum on modifiable risk factors for falls in an aging population, and our assessment methods will be designed to reflect this important outcome.

### Limitations

Our community-university partnership produced an appealing, enjoyable, and impactful recreation program. However, we must acknowledge some limitations. The small-scale pilot nature of our study and the lack of a control group prevent our ability to draw inferences of causation. Furthermore, several confounding factors in the intervention may have influenced our positive findings, including the socially engaging nature of the program alone and interaction with program instructors. It is also possible that increases in participants’ leisure-time physical activity outside of the program could have contributed to our favorable results. While participants were instructed to maintain their same activity habits, leisure-time physical activity was not monitored during the program.

Also, regarding the training sessions, participants received 1 hour of exercise and 1 hour of art each week, which is a relatively low volume of exposure. While we are encouraged by our findings that even a low dose of training was associated with functional improvements, we do not know the impact that a higher dose of recreational activities would have on the same outcomes. Future studies might be designed with different doses of training to identify which is optimally beneficial for a variety of outcomes.

In addition, a limited sample size restricts us from developing predictive models or exploring the impacts of possible covariates. Future studies will focus on expanding the inclusion of more community-based sites to increase sample size. In addition, we were only able to enroll female participants, which is an ongoing challenge in this community setting. Future interventions will use more targeted recruitment strategies to improve diversity in gender and race or ethnicity. This includes enhancing awareness of our program in the community through a series of open-house events to showcase the program activities. We also propose working directly with older men in the community to develop a training program that is more appealing to them. In addition, while our instructors at each site received identical training, there may have been a small variation in content delivery between YMCA branches. However, we did not integrate any implementation outcomes, such as fidelity to track these factors.

We must also acknowledge that the combination of exercise and art prevents us from exploring the impact of the individual components on our program. This would require at least three groups: (1) combined exercise and art, (2) exercise only, and (3) art only. However, it must be noted that the purpose of our study was to elucidate the therapeutic benefits of the full recreation program and not of the individual components. However, we theorized that inclusion of the artmaking would enhance participant engagement, create more opportunities for mastery and skill-building, build self-confidence, and create a more enjoyable overall experience. In addition, we did not include assessments of fine motor skills to better characterize the benefits of the art-making component. As stated above, parsing out the individual effects of the art classes would require a different study design, but would be valuable. Inclusion of a more diverse selection of functional outcomes could potentially answer additional questions about the real-world impact of our program.

Upon examination of our major quantitative findings in Table 3, we observe several effect sizes that might be unexpectedly large, given the small sample size. To derive the effect size, we relied on the “Exact” method given the sample size of 17, which is appropriate. However, we must emphasize the exploratory nature of our findings and encourage the reader to examine them with caution. Future studies will enroll a larger sample to more deeply explore these associations.

In addition, we observed similar changes to our previous study [12] in the Stroop IS. However, we must also interpret this with caution. The IS includes performance on both the word and color scores of the test. Improvement in either of these 2 conditions will produce a higher IS. In this study, we saw improved scores on the incongruent condition, and therefore, are hopeful that our program helped improve interference inhibition in this population.

The purpose of this pilot was to leverage the power of our partnership with the Buffalo-Niagara YMCA to implement a group recreation program that fosters social support and helps preserve cognitive and physical function. From this small pilot, we have developed a larger-scale program involving four YMCA branches and an attention-control group that we anticipate will allow us to more deeply explore the previously described associations and generate a more comprehensive explanatory model. This first pilot of the Movers and Makers program served as an effective stepping stone, allowing us to gather key insights from community members, develop and test our implementation methods, and assess limitations.

### Comparison With Previous Work

In examining the existing literature, we have not found any studies that have specifically assessed the implementation and impacts of group-based, SMARTfit exergaming and art making on cognitive and physical function in older adults in a community setting. However, several randomized controlled trials have sought to characterize the effects of combined creative activities and exercise in general on cognition, well-being, quality of life, depressive symptoms, and subjective health outcomes [38-41].

Fundamentally, when we examine the different populations that have been studied in the existing literature, we observe important differences. Our study enrolled community-dwelling older adults, 65 years or older, with at most MCI. Kamegaya et al [38] recruited community-dwelling older adults at risk for cognitive decline without apparent dementia. In this study, a 12-week intervention consisting of aerobic exercise, stretching, and walking, along with games, crafts, and cooking, improved cognition and well-being [38]. In this study, the intervention duration was also 12 weeks, and we also found improvements in cognition, in a similar study population, that did not have overt dementia. Furthermore, Roswiyani et al [41] used a 4-group design (art-only, Qigong-only, art plus Qigong, and control) and 2 training sessions per week for 8 weeks. Results show improved well-being (social relations) among adults (age ≥50 y) assigned to the art-only group. Furthermore, depressive symptoms also declined in the art-only group and in the art plus Qigong group. The Qigong-only group did not demonstrate improvements in any functional outcomes [41]. While the populations among these studies are similar, important differences can be observed in the modes of exercise and art being delivered, with activities ranging from single-task training, like walking [38], to the more complicated dual-task challenges tested in our intervention. This high degree of variability has made it very difficult to synthesize the overall findings of similar studies across the literature.

Other investigators have examined these associations in individuals with dementia. Kang et al [39] explored the effects of a 9-week intervention that consisted of two 3-hour sessions each week, of cognitive stimulation, music, art, horticulture, and exercise. Participants received 30 minutes of cognitive stimulation, followed by 30 minutes of music therapy, a 10-minute break, then 30 minutes of art therapy, 30 minutes of a horticultural activity, and 30 minutes of memory training. Participation in this program among 20 older adults with dementia was associated with improved depression levels, mental-emotional health, and cognitive function. Similarly, Viola et al [40] implemented a 12-week intervention to assess the impact of memory training, computerized cognitive stimulation, writing, and painting, along with walking and stretching on patients with mild Alzheimer disease. These investigators found that participation was associated with improved quality of life and depressive symptoms. Cognitive function did not change in response to participation in the intervention [40]. Differences between these studies can be seen in their effect on cognition, with no improvements found in the study by Viola et al [40]. This highlights an important methodological difference that likely impacts the variability in outcomes we have observed. To date, the optimal dose of exercise and cognitively stimulating activities like creative pursuits that improve cognitive and physical functional outcomes has not been identified. Larger studies are still needed to fully explore these complex associations.

In comparison with previous studies that explore the effects of exercise and creative activities on health and function in aging, our study has some unique attributes. Our study appears to be the first with this focus to include key principles of community-based research to develop and test our intervention activities. Program design and implementation were directly influenced by feedback from older adult community members, creating a more appealing and relevant program that encouraged participation. Our study also integrated a qualitative assessment of the participants’ experiences in the intervention program that is being used to design future larger-scale studies. The insights provided by participants have been invaluable in increasing the appeal, usability, and relevance of the program activities.

### Conclusions

Our Movers and Makers program is among the first initiatives to combine group-based exergaming and guided artmaking in a community setting to improve key cognitive and physical functional outcomes in older adults. The YMCA’s dedication to promoting successful aging makes it an ideal partner in developing and implementing this novel program. Our team was able to create a highly enjoyable and satisfying program, leading to a completion rate of 100%. The participants’ positive experience in the program was impacted by the immersive, enriching nature of each activity (exergaming and art) and the high quality of instruction. Through this unique collaboration, we were able to assemble a highly effective interdisciplinary team that was genuinely dedicated to the success of the Movers and Makers program. Team members created a supportive and caring environment that was energizing and inspiring. Furthermore, our approach that emphasized creating a socially engaging atmosphere likely contributed to participants’ willingness to complete the program.

We employed a community-integrated approach to designing and implementing our program, which produced an appealing and impactful training experience that is adaptable to meet the needs of different populations in different environments. Future efforts must focus on assessing key implementation outcomes in larger-scale interventions that integrate a control group. These changes will allow our team to generate a more comprehensive explanatory model and identify potential barriers to optimally delivering the Movers and Makers curriculum in diverse community settings with unique strengths and limitations.

## Abbreviations

30STS: 30-second Sit-to-Stand test
6MWT: 6-Minute Walk Test
FSBT: 4-Stage Balance Test
IS: interference score
KTY: Ken-Ton Family YMCA
MCI: mild cognitive impairment
MoCA: Montreal Cognitive Assessment
Pcw: predicted color-word score
SCWT: Stroop Color and Word Test
TMT: Trail-Making Test
WEY: William-Emslie Family YMCA
